# A Call from New Jersey

**Published:** 1900-11

**Authors:** Foster M. Voorhees

**Affiliations:** State of New Jersey, Executive Department, Trenton


### A CALL FROM NEW JERSEY.

STATE OF NEW JERSEY, EXECUTIVE DEPARTMENT,
TRENTON, October 11, 1900.

DR. WILLIAM HERBERT LOWE:

MY DEAR DOCTOR: I have your letter of September 18th in the matter of the thirty-eighth annual convention of the American Veterinary Medical Association, and note its contents.

As Governor of this State, I do most heartily extend to the above Association an invitation to make Atlantic City, in this State, the meeting place of its annual convention for 1901.

I do not believe your Association can find anywhere a place better adapted for its work and for the care of the representatives who may be present, and I do most heartily hope your efforts in influencing your Association to select Atlantic City will meet with success.

Very truly yours,
FOSTER M. VOORHEES.

---

The unanimous vote of the Veterinary Medical Association of New Jersey, at its meeting in Atlantic City on September 13th, inviting the American Veterinary Medical Association to that city in 1901, was followed by a strong petition from the Pennsylvania State Association at Erie on the 18th, urging the Executive Committee of the American body to respond to the wishes of our New Jersey colleagues.

A meeting of the Missouri Valley Veterinary Medical Association was held at Kansas City, Missouri, October 29, 1900.

The next regular meeting of the Veterinary Medical Association of New Jersey will be held at Trenton, January 10, 1901.

The next meeting of the South Dakota State Veterinary Medical Association, January, 1901.

